# DNMT3B-579G>T (rs1569686G>T) polymorphism and the risk of multiple sclerosis in a subset of Iranian population

**Published:** 2019-04-04

**Authors:** Nasrin Yazdanpanahi, Masoud Etemadifar, Elaheh Shams

**Affiliations:** 1 Department of Biology and Genetics, Falavarjan Branch, Islamic Azad University, Isfahan, Iran; 2 Department of Neurology, School of Medicine, Isfahan University of Medical Sciences, Isfahan, Iran; 3 Isfahan Multiple Sclerosis Research Center, Isfahan, Iran; 4 Young Researchers and Elite Club, Falavarjan Branch, Islamic Azad University, Isfahan, Iran

**Keywords:** Multiple Sclerosis, Genes, DNA Methyltransferase, Polymorphism, Genetic, Iran

## Abstract

**Background:** Deoxyribonucleic acid (DNA) methyltransferase 3 beta (DNMT3B) gene encodes an MT enzyme involving in de novo methylation of DNA. The present investigation aimed to explore the association of DNMT3B-579G>T (rs1569686) polymorphism with multiple sclerosis (MS).

**Methods:** 130 Iranian patients with MS and 130 controls were genotyped for the DNMT3B-579G>T using polymerase chain reaction-restriction fragment length polymorphism (PCR-RFLP) method.

**Results:** There was no statistically significant association between DNMT3B-579G>T and susceptibility to MS. The alleles and genotypes of DNMT3B-579G>T did not have different risks of MS development under various models [T vs. G (P = 0.86); GTvs. GG (P = 0.48); TT vs. GG (P > 0.99); GT+TT vs. GG (P = 0.60), and TT vs. GG+GT (P = 0.87)]. Also, there was no statistically significant association between genotypes and clinical and demographic characteristics of patients (P > 0.05).

**Conclusion:** The current findings suggest that DNMT3B-579G>T is probably not a crucial potential risk marker in molecular diagnostics of MS among Iranian. However, to the best of our knowledge, this is the ﬁrst genetic association study about the DNMT3B polymorphisms and MS. Therefore, further surveys should be included to estimate the exact relevance of DNMT3B gene to the development of autoimmune disorders like MS.

## Introduction

Multiple sclerosis (MS) is a complex autoimmune disease associated with inflammation, demyelination, and degeneration of neurons.

The disease usually affects young people and therefore, can have socio-economical impacts on population. This effect, along with an increasing worldwide incidence of MS made it a crucial public health problem.^1^ According to different epidemiological studies, genetic predisposition and non-genetic influence play a role in the pathogenesis of this disease.^2^^-^^4^ Epigenetics, as a bridge between genotype and phenotype, is a relatively new area of MS research. Environmental risk factors can exert their effects on gene expression and in consequence on phenotype through epigenetic modifications. Aberration in epigenetic mechanisms may contribute in the development and clinical symptoms of MS. The maternal origin effect in MS relating to imprinting and X-chromosome inactivation, the effect of major environmental risk agents of MS on epigenetic mechanisms, existence of modified histone in non-lesioned white matter of patients with MS, and the low concordance rate of identical twins, all are evidence suggesting that epigenetics may have a role in pathogenesis of MS.^5^^,^^6^

Deoxyribonucleic acid (DNA) methylation is one of the important mechanisms for epigenetic modifications and is under the control of DNA methyltransferases (DNMTs). The family of DNMTs includes five different enzymes (DNMT1, DNMT2, DNMT3A, DNMT3B, and DNMT3L) and incorporates methyl group to the C5 of cytosine to form the 5-methylcytosine (5mC). Hypermethylation and hypomethylation of DNA leads to repression and activation of genes, respectively, through the changes of chromatin structure.^7^ DNA hypomethylation is associated with activation, differentiation, and commitment of immune cells.^8^

To date, most studies have been conducted about the association of DNMT genes with cancer, but the number of investigations about the role of these genes in pathogenesis of autoimmune disorders is few. There is, however, growing evidence about the relationship between methylation aberration or impaired DNMTs function and autoimmune diseases such as systemic lupus erythematosus (SLE), rheumatoid arthritis (RA), Sjogren's syndrome (SS), MS, psoriasis, and autoimmune thyroid diseases (AITD).^6^^,^^9^^,^^10^

DNMT3B gene has been mapped on chromosome 20q11.2 and encodes a DNMT enzyme contributing mainly in de novo methylation.^7^ Single nucleotide polymorphisms (SNPs) in this gene could affect the potential of DNA methylation, gene expression, and consequently, the development of various diseases. In the current study, the association of DNMT3B-579G>T polymorphism with MS has been studied. This polymorphism is a common SNP in the promoter region of DNMT3B with a controversial role in gene expression. To the best of our knowledge, this is the first investigation about the relevance of DNMT3B gene polymorphism with MS.

## Materials and Methods

Research Ethics Committee of Najaf Abad Branch, Islamic Azad University, Isfahan, Iran, approved the investigation (approval number: IR.IAU.NAJAFABAD.REC.1396). Also, a questionnaire and written informed consent was obtained from all participants. 

In the present case-control study, a total of 260 Iranian individuals including 130 patients with unrelated relapse remitting MS (RRMS) and 130 age- and sex-matched controls were selected. All patients were selected from Isfahan MS Research Center and were diagnosed as cases of RRMS.

The diagnosis of patients with MS was conducted by an expert neurologist, and was based on clinical examinations and the demonstration of neurologic signs subsequent to white matter lesions. To distinguish MS from other similar neurologic diseases, McDonald criteria^11^ were evaluated using adjuvant laboratory tests and imaging, including magnetic resonance imaging (MRI) of brain and spinal cord, cerebrospinal fluid (CSF) analysis, and functional assays of the nervous system. The diagnosis and confirmation of MS was largely based on the results of MRI test. All controls were also diagnosed with no autoimmune diseases including MS by a neurologist and had no clinical or laboratory evidence of the diseases according to medical records. 

***DNA extraction:*** The genomic DNA extraction was done using 500 μl peripheral blood applying PrimePrep Genomic DNA Isolation Kit (GeNet Bio, Korea) according to the manufacturer's guidance.

***Polymerase chain reaction-restriction fragment length polymorphism (PCR-RFLP):*** The extracted DNA was assesed for quality and quantity using agarose gel electrophoresis and spectrophotometry. 

PCR-RFLP method was used for genotype analysis of DNMT3B-579G>T. PCR was performed in a final standard volume of 25 μl containing 0.5 μl of the primers (10 pM) (Forward: GTGACCTGGAGCTGTTTGTG, Reverse: GCAACAACCTGTATCTCCCC), 2 μl magnesium chloride (MgCl_2_) (50 mM), 0.5 μl deoxynucleoside triphosphate (dNTP) mix (10 mM), 2.5 μl Taq PCR buffer (10X), 0.1 μl Taq DNA polymerase (5 u/μl), 2 μl DNA (50 ng), and 16.9 μl double-distilled water (ddH_2_O). 

The PCR condition was as follows: initial denaturation at 94 °C for 5 minutes, followed by 36 cycles of 94 °C for 40 seconds (denaturation), 58 °C for 35 seconds (annealing), 72 °C for 35 seconds (extension), and final extension at 72 °C for 10 minutes. 

PCR with the above mentioned condition was applied to amplify a 296 bp length fragment including a -579G>T polymorphic site. The entire PCR product was digested by 2.5 units of PvuII restriction enzyme (Fermentas, Germany). The obtined fragments were separated using electrophoresis on 2.5% agarose gel for 1 hour at 110 V and bands were visualized by green viewer dye under ultraviolet (UV) light.

**Figure 1:**
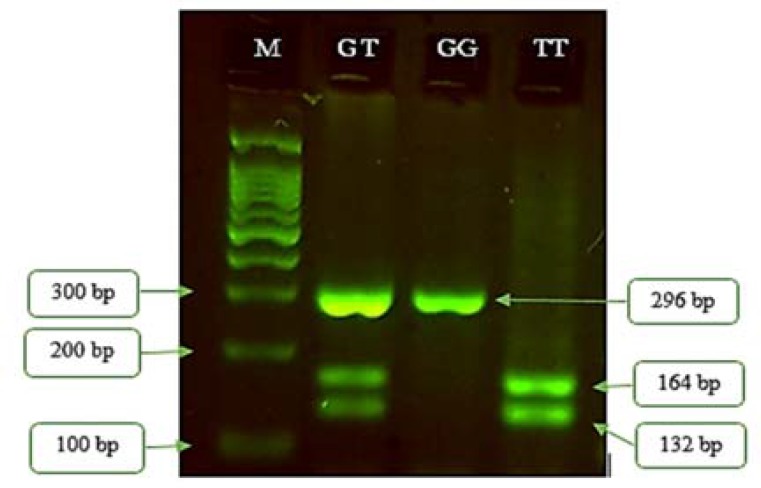
Gel electrophoresis of polymerase chain reaction-restriction fragment length polymorphism (PCR-RFLP) product. M: ladder (100 bp), GT: heterozygote for G and T alleles, GG: homozygote for G allele, TT: homozygote for T allele

The G allele lacks PvuII restriction site and therefore, results in a single 296 bp fragment after PCR-RFLP. The T allele introduces the PvuII cutting site and so, digestion of this allele with the enzyme produces two fragments (132 and 164 bp). Thus, RFLP assay showed the following patterns: A single 296 bp fragment for G/G genotype, 132 and 164 bp fragments for T/T genotype, and 132, 164, and 296 bp fragments for G/T genotype (Figure 1). 

All statistical analyses were conducted applying SPSS software (version 20, IBM Corporation, Armonk, NY, USA). The results were expressed as mean ± standard deviation (SD). The Hardy-Weinberg equilibrium (HWE) was evaluated using the chi-square (χ^2^) test. Also, χ^2^, Fisher’s exact test, and ANOVA were applied to compare the frequency distribution of genotypes, alleles, and demographic features between groups. Odds ratio (OR) and 95% confidence interval (CI) were employed for the evaluation of risk factors. P-values ≤ 0.05 were considered as statistical significance.

## Results

***Clinical and demographic characteristics:*** 130 patients with RRMS (mean age: 33.04 ± 7.91 years, range:16-50) and 130 controls (mean age: 31.72 ± 6.68 years, range:18-45) were entered in this study. The number of men was 28 (21.5%) and 33 (25.4%) in patients and controls, respectively. Cases and controls did not have significant difference in terms of age and sex (P = 0.15 and P = 0.56, respectively). 

The genotypic distribution of the DNMT3B-579G>T polymorphism was in HWE for the two study groups (χ^2^ = 0.40, P = 0.82 and χ^2^ = 0.34, P = 0.84, respectively).

The findings of the current investigation suggest no relationship between MS susceptibility and DNMT3B-579G>T polymorphism in the studied Iranian population according to table 1.

Allelic and genotypic distributions of the mentioned polymorphism in patients and controls have been shown in table 1. The alleles and genotypes of DNMT3B-579G>T did not have different risks of MS development under various models (OR _T__vs. G_ = 0.95, 95% CI = 0.67-1.35, P = 0.86; OR _GT vs. GG_ = 0.80, 95% CI = 0.46-1.38, P = 0.48; OR _TT vs. GG_ = 0.97, 95% CI = 0.48-1.96, P > 0.99; OR _GT+TT vs. GG_ = 0.84, 95% CI = 0.50-1.41, P = 0.60; and OR _GG+GT vs. TT_ = 1.11, 95% CI = 0.59-2.07, P = 0.87, respectively). 

Also, there was no significant association between DNMT3B-579G>T genotypes and age of onset (P = 0.79), sex (P = 0.13), disease duration (P = 0.35) and Expanded Disability Status Scale (EDSS) (P = 0.20) in patients (Table 2). The result of gel electrophoresis of RFLP has been shown in figure 1.

## Discussion

So far, many efforts have been conducted to study the genetic architecture of MS in different populations like Iran.^4^^,^^12^^-^^15^

**Table 1:** Genotype and allele frequencies of DNMT3B-579G>T in controls and cases

**Variables**	**Controls [n (%)]**	**Cases [n (%)]**	**P**	**OR (95% CI)**
**Genotypes**				
GG	40 (30.8)	45 (34.6)	-	1
GT	67 (51.5)	60 (46.2)	0.48	0.80 (0.46-1.38)
TT	23 (17.7)	25 (19.2)	> 0.99	0.97 (0.48-1.96)
GG	40 (30.8)	45 (34.6)	-	1
TT+GT	90 (69.2)	85 (65.4)	0.60	0.84 (0.50-1.41)
GG+GT	107 (82.3)	105 (80.8)	-	1
TT	23 (17.7)	25 (19.2)	0.87	1.11 (0.59-2.07)
Alleles				
G	147 (56.5)	150 (57.7)	-	1
T	113 (43.5)	110 (42.3)	0.86	0.95 (0.67-1.35)

OR: Odds ratio; CI: Confidence interval

However, these attempts have only been able to identify a small proportion of the causes of disease, highlighting the need to assess non-genetic factors as well as the interplay between environmental and genetic factors. In addition, genetic effect alone does not justify the low MS concordance rate between monozygotic twins. In recent years, different studies have provided evidence about the connection of epigenome and autoimmune diseases like MS.^6^^,^^10^ DNMT3B protein is one of the MT enzymes creating a new methylation pattern on DNA by de novo methylation.^7^ The common SNP of this gene, -579G>T, is a G to T transition in promoter (-579 bp from exon 1B transcription site).^16^ The functional role of the DNMT3B-579G>T polymorphism is controversial. According to some investigations, it may affect promoter activity and gene expression levels, alone or in combination with other DNMT3B polymorphisms. The results of these studies suggested the association between DNMT3B-579G>T and different diseases.^17^^-^^20^

 Various studies have provided evidence for the association of DNMT3B polymorphisms with cancers. According to different meta-analyses, DNMT3B-579G>T might involve in the susceptibility to cancers, especially in Asian populations. The findings of these investigations suggested that the DNMT3B-579T allele was associated with an increased risk of cancer.^17^^,^^19^

The correlation of DNMT3B-579G>T polymorphism with gastric cancer has been identified in different ethnic groups. The results of a study conducted in a subset of Iranian patients with gastric cancer suggested that the DNMT3B-579T allele might increase the relative risk for the progression of clinicopathological characteristic of tumor grade in affected individuals.^21^ Also, this allele showed a significant difference between patients with gastric cancer and control group in population of China.^22^ There was no significant relationship between DNMT3B-579G>T and gallbladder carcinoma in Indian population.^23^ Moreover, the DNMT3B-579G>T polymorphism may affect the susceptibility to lung and colorectal cancers in Chinese population. Individuals with TT genotype indicated a higher risk of lung and colorectal cancers compared to those who had other genotypes.^24^^,^^25^

**Table 2:** Clinical and demographic characteristics of patients stratified according to DNMT3B-579G>T genotypes

**Characteristics**	**Genotypes**			**P**
	**GG**	**GT**	**TT**	
Age (year) (mean ± SD)	33.64 ± 7.47	32.17 ± 8.42	34.04 ± 7.49	0.50
Sex [n (%)]				0.13
Male	13 (28.9)	13 (21.7)	2 (8.0)	
Female	32 (71.1)	47 (78.3)	23 (92.0)	
Age of onset (year) (mean ± SD)	28.58 ± 7.54	27.63 ± 7.95	28.56 ± 7.86	0.79
Disease duration (year) (mean ± SD)	5.07 ± 3.10	4.53 ± 2.55	5.48 ± 3.24	0.35
EDSS (mean ± SD)	1.93 ± 0.87	1.88 ± 0.92	2.26 ± 0.93	0.20

SD: Standard deviation

Also, DNMT3B-579G>T did not show a significant association with Head and neck squamous cell carcinoma (HNSCC) in non-Hispanic whites. However, TT genotype of this polymorphism was linked to the increased risk of SCCHN when -579G>T was analyzed in combination with the other polymorphism of DNMT3B (-149C>T; rs2424913).^26^

In another investigation, no association was revealed between the polymorphisms of DNMT3B and ovarian cancer in Polish women.^27^

 However, a few investigations have studied the relationship of DNMT3B polymorphisms with autoimmune disease**. **The T allele and TT genotype of DNMT3B-579G>T were associated with increased risk of developing thymoma in Italian patients with myasthenia gravis (MG). However, no association was detected in MG patients without thymoma.^28^

There was no significant relationship between DNMT3B-579G>T and idiopathic thrombocytopenic purpura (ITP) in a Chinese population.^29^ In contrast, -579 TT genotype was linked to increased risk of this disease in Egiptian children.^18^

Furthermore, DNMT3B-149C>T polymorphism was associated with AITDs^30^ but not with primary gout arthritis in Chinese population.^31^ The relationship between DNMT3B and MS was also identified recently. This study suggested that messenger ribonucleic acid (mRNA) levels of DNMTs including DNMT3B were increased and demethylation enzymes were lower in demyelinated MS hippocampus than those in myelinated MS hippocampus. Comparative methylation profiling revealed hypomethylation within upstream sequences of 6 genes and hypermethylation of 10 genes in demyelinated hippocampus of patients with MS. The increase of DNMTs including DNMT3B expression could involve in methylation of target genes. Independent validation by reverse transcription PCR (RT-PCR) showed that DNA methylation inversely was associated with mRNA levels of the candidate genes.^32^

The present investigation is the first study about the association of DNMT3B polymorphism and MS that evaluated the relationship between -579G>T polymorphism and this disease. 

The current results did not show any association between genotype or allele frequency of DNMT3B-579G>T and MS. The frequency of G allele and minor allele, T, was 56.5% and 43.5%, respectively, among controls. This result is almost in agreement with the findings of a previous study which investigated the association of DNMT3B-579G>T polymorphism with gastric cancer in Iranian population (a frequency of 60.71% and 39.29% for G allele and minor allele, T, respectively, among controls).^21^ These results also were relatively consistent with those revealed in European population (a frequency of 65.0% and 35.0% for G and T alleles, respectively, among controls).^20^^,^^27^ However, the results of the present study were different from those observed in other Asian populations with a higher frequency of T allele than G allele for DNMT3B-579G>T polymorphism.^23^^,^^24^^,^^33^ The dissimilarity between the ethnicity and size of samples might be causes for the differences of allele and genotype distribution shown in the current study from those reported in previous investigations. 

## Conclusion

According to the current research, DNMT3B-579G>T polymorphism does not seem to represent a major predictive marker for MS, at least in Iranian population. However, as this is the first study about the association of DNMT3B-579G>T with MS, for unraveling the exact relationship between DNMT3B and its polymorphisms with MS, further investigations considering the effect of mentioned polymorphism in relation with other genetic or environmental agents are needed. Moreover, investigation of different ethnic groups and also samples with a larger size would be helpful to gain further conclusive results.

## Notes:

**How to cite this article:** Yazdanpanahi N, Etemadifar M, Shams E. DNMT3B-579G>T (rs1569686G>T) polymorphism and the risk of multiple sclerosis in a subset of Iranian population. Iran J Neurol 2019; 18(2): 70-5.
